# Relationship of gender and age on thyroid hormone parameters in a large Chinese population

**DOI:** 10.20945/2359-3997000000179

**Published:** 2019-09-25

**Authors:** Xinxin Chen, Xulei Zheng, Zhaojun Ding, Yang Su, Shu Wang, Bin Cui, Zhen Xie

**Affiliations:** 1 Ruijin Hospital Shanghai Jiao Tong University School of Medicine Shanghai China Clinical Center for Endocrine and Metabolic Diseases, Institute of Endocrine and Metabolic Diseases, Ruijin Hospital, Shanghai Jiao Tong University School of Medicine, Shanghai, China; 2 Shanghai Xuhui Central Hospital Zhongshan-Xuhui Hospital Fudan University Shanghai China Department of Endocrinology, Shanghai Xuhui Central Hospital/Zhongshan-Xuhui Hospital, Fudan University, Shanghai, China; 3 Clinical Laboratory Sichuan Provincial People's Hospital University of Electronic Science and Technology of China Chengdu China Clinical Laboratory, Sichuan Provincial People's Hospital, University of Electronic Science and Technology of China, Chengdu, China; 4 Chinese Academy of Sciences Sichuan Translational Medicine Research Hospital Chengdu China Chinese Academy of Sciences Sichuan Translational Medicine Research Hospital, Chengdu, China; 5 Department of Dermatology Sichuan Provincial People's Hospital University of Electronic Science and Technology of China Chengdu China Department of Dermatology, Sichuan Provincial People's Hospital, University of Electronic Science and Technology of China, Chengdu, China

**Keywords:** Thyroid hormone, thyrotropin (TSH, Gender and age, Chinese population

## Abstract

**Objective:**

This study aimed to present the impact of age and gender on thyroid hormone levels in a large Chinese population with sufficient iodine intake.

**Subjects and methods:**

A total of 83643 individuals were included and were stratified by age and gender. The median, 2.5th and 97.5th of thyrotropin (TSH), free triiodothyronine (FT3), free thyroxine (FT4) and FT3/FT4 ratio were calculated for both genders for every decade from 18 to over 80 years. TSH, FT3, FT4, FT3/FT4 distribution in each age group was evaluated for females and males using smoothing splines in the generalized additive models (GAM). TSH concentrations were compared in the different age groups in gender.

**Results:**

In the over 80s age group, the TSH level (median: 2.57 mIU/L, 2.5th-97.5th: 0.86-7.56 mIU/L) was significantly higher than other age groups, irrespective to gender (P<0.001). Females had a higher TSH value than males in all age groups (P<0.001). Results of the smoothing curves showed that TSH increased with age, FT3 concentration was higher in males than in females and the tendency of the FT3/FT4 ratio was basically similar to that of FT3. TSH concentration in the 50s age group (median 2.48 mIU/L for females versus 2.00 mIU/L for males) was significantly higher than that in the 30s age group (median 2.18 mIU/L for females versus median 1.85 mIU/L for males).

**Conclusions:**

In accord with increasing TSH values during aging, females and older adults have lower FT3 values and lower FT3/FT4 ratios, while the FT4 values remain stable.

## INTRODUCTION

A growing number of studies have shown that the hypothalamic-pituitary-thyroid axis response to thyroxine (T4) and triiodothyronine (T3) levels has changed due to modifications in the set points by age (1,2). The thyroid gland affected by age might modify the set point of response to FT4 or FT3 level, resulting in changed thyrotropin (TSH) levels in older adults (3,4). Surks and cols. (5) reported that within the age-specific distribution of serum TSH in the US population, the 97.5th percentiles were 5.9 mIU/L for individuals aged 70-79 and 7.5 mIU/L for those aged 80 and older. Zhai and cols. (6) examined the prevalence of thyroid disease in older adults aged ≥ 65 years and found that the serum TSH level increases with age, which may represent a normal compensatory phenomenon in that age group. The evidence from such studies suggests that TSH levels increase with age and that an age-specific reference limit for TSH is essential.

However, the idea of age-related changes in TSH remains controversial and few studies have focused on the age-related changes in thyroid hormone in large Asian populations. In China, an epidemiological study of 5348 inhabitants living in areas with sufficient iodine indicated that females had a higher TSH value than males, but no positive correlation was detected between age and TSH values (7). Biochemically manifested thyroid dysfunction with abnormalities in serum T4, T3 and thyrotropin (TSH) levels has a higher prevalence in females (5- to 10-fold higher) than in males (8-10). Therefore, the association between TSH levels and age and gender are of great importance in diagnosing and treating thyroid disease, especially in the subclinical thyroid disease stages (11).

The goal of the present study was to represent the impact of age and gender on the levels of thyroid hormone. Specifically, this study aimed to investigate the relationship between thyroid hormone and age/gender in a large Chinese population of 83643 individuals aged in 18-90 years old who live iodine sufficient areas.

## SUBJECTS AND METHODS

### Study population

This was a retrospective analysis of the patients’ data. The participants with thyroid function tests were recruited in physical examination center of Sichuan Provincial People’s Hospital from October 2013 to April 2017.We identified participants as having no thyroid disease using the following exclusion criteria: 1) adults without a diagnosed thyroid disease; 2) serum FT4 or FT3 not within the reference range (FT4 9.01–19.05 pmol/L and FT3 2.63–5.70 pmol/L); 3) serum TSH levels lower than 0.35 mIU/L or above 10 mIU/L, since such patients would have been under medical supervision and were more likely to be ill (12); 4) to eliminate the influence of thyroid antibody, population with positive thyroid antibody was excluded. Positive thyroid antibody was defined as serum TgAb > 75 IU/mL and serum TPOAb > 30 IU/mL.

Serum TSH, FT4 and FT3, thyroid peroxidase antibodies (TPOAb), thyroglobulin antibodies (TgAb) and related information such as gender and age were included in this study. The study was approved by the ethics committee in Sichuan Provincial People’s Hospital.

### Laboratory assays

Standard laboratory quality evaluation procedures were routinely employed, and regular participation at interlaboratory tests was also part of the quality management strategy. Serum levels of TSH, FT3, FT4, TgAb, and TPOAb were measured using automated chemiluminescent immunoassays (Architect i2000SR; Abbott Laboratories, Chicago, IL). The functional sensitivity of serum TSH was 0.0036 mIU/L. The laboratory reference ranges provided by the manufacturer were as follows: TSH 0.35–4.94 mIU/L, FT4 9.01–19.05 pmol/L, FT3 2.63–5.70 pmol/L, TPOAb < 30 IU/ml, and TgAb < 75 IU/ml.

### Statistical methods

Statistical analyses and screening for all figures were obtained using R version 3.5.1, as previously described (13). The euthyroid participants were stratified by age groups as follows: 18-29, 30-39, 40-49, 50-59, 60-69, 70-79, age 80 years and older (“over 80s group”). The median, 2.5th percentiles, 97.5th percentiles of TSH, FT3, FT4 were calculated for each age group. Wilcoxon scores (rank sums) and Kruskal-Wallis test were used to compare the nonparametric TSH distributions of the different age groups. The median of TSH, FT3, FT4 and FT3/FT4 ratio was plotted using smoothing splines in generalized additive models (GAM) (14). The frequency distribution curves of TSH concentration were compared between the 30s and 50s age groups according to TSH distributions at all ages.

## RESULTS

In total, the data of 97619 participants were analyzed in the present study. After excluding participants with abnormal FT4, 12.97% (12661) participants were positive for the thyroid antibody, and 0.35% (300) participants had TSH levels higher than 10 mIU/L. Finally, a total of 83643 participants, including 48602 (57.5%) males and 35509 (42.5%) females, were in the further analyses. As shown in Table 1, the study population was divided into age groups of 18-29 years (n = 13303, 15.9%), 30-39 years (n = 20764, 24.8%), 40-49 years (n = 23278, 27.8%), 50-59 years (n = 15112, 18.1%),60-69 (n = 7180, 8.6%),70-79 (n = 2826, 3.4%), and age 80 years and older (n = 1180, 1.4%). Most of the participants were younger than age 50 years and a relatively large portion was within the 30-50 year age range.

**Table 1:** TSH, FT3, FT4 and FT3/FT4 ratio on Median, 2.5th and 97.5th percentiles by gender and age in non-thyroid disease participants

Age-group	Number N	TSH (mIU/L) Median (2.5th-97.5th)	FT3 (pmol/L) Median (2.5th-97.5th)	FT4 (pmol/L) Median (2.5th-97.5th)	FT3/FT4 ratio Median (2.5th-97.5th)
**Total**					
18~29	13303	2.04 (0.79-5.17)	4.70 (3.57-5.62)	12.93 (10.50-15.82)	0.36 (0.27-0.46)
30~39	20764	1.98 (0.77-5.20)	4.64 (3.55-5.61)	12.72 (10.37-15.60)	0.36 (0.27-0.47)
40~49	23278	2.08 (0.76-5.68)	4.61 (3.52-5.60)	12.55 (10.14-15.62)	0.37 (0.27-0.47)
50~59	15112	2.16 (0.77-5.99)	4.62 (3.56-5.57)	12.52 (10.06-15.62)	0.37 (0.27-0.47)
60~69	7180	2.17 (0.74-6.16)	4.57 (3.49-5.54)	12.55 (10.00-15.70)	0.36 (0.27-0.47)
70~79	2826	2.30 (0.79-6.71)	4.41 (3.34-5.41)	12.68 (9.86-16.18)	0.35 (0.25-0.46)
≥ 80	1180	2.57 (0.86-7.56)	4.18 (3.09-5.06)	12.77 (9.97-16.25)	0.33 (0.23-0.43)
**Male**					
18~29	6397	1.90 (1.39-4.65)	4.88 (4.53-5.65)	13.03 (12.11-16.04)	0.37 (0.34-0.47)
30~39	11554	1.85 (1.35-4.67)	4.82 (4.48-5.64)	12.79 (11.88-15.7)	0.38 (0.34-0.48)
40~49	13776	1.91 (1.38-4.94)	4.77 (4.42-5.63)	12.59 (11.65-15.69)	0.38 (0.34-0.48)
50~59	9166	1.99 (1.41-5.34)	4.70 (4.35-5.60)	12.49 (11.57-15.60)	0.37 (0.34-0.48)
60~69	4425	2.02 (1.45-5.43)	4.61 (4.23-5.56)	12.44 (11.45-15.60)	0.37 (0.33-0.48)
70~79	1891	2.21 (1.61-6.62)	4.41 (4.04-5.41)	12.58 (11.50-16.13)	0.35 (0.31-0.47)
≥ 80	853	2.50 (1.72-7.15)	4.18 (3.82-5.06)	12.65 (11.68-16.25)	0.33 (0.30-0.43)
**Female**					
18~29	6906	2.20 (1.57-5.51)	4.53 (4.18-5.49)	12.84 (12.01-15.60)	0.35 (0.32-0.45)
30~39	9210	2.19 (1.57-5.64)	4.40 (4.06-5.37)	12.62 (11.79-15.43)	0.35 (0.32-0.44)
40~49	9502	2.40 (1.70-6.40)	4.38 (4.04-5.41)	12.49 (11.64-15.49)	0.35 (0.32-0.45)
50~59	5946	2.46 (1.73-6.84)	4.5 (4.15-5.47)	12.56 (11.71-15.64)	0.36 (0.32-0.46)
60~69	2755	2.43 (1.70-6.96)	4.52 (4.18-5.50)	12.71 (11.73-15.89)	0.35 (0.32-0.46)
70~79	935	2.51 (1.59-7.20)	4.39 (4.03-5.40)	12.91 (11.90-16.32)	0.34 (0.31-0.44)
≥ 80	255	2.90 (2.00-8.03)	4.20 (3.85-5.13)	13.19 (11.95-16.47)	0.32 (0.28-0.44)

**Figure 1:**
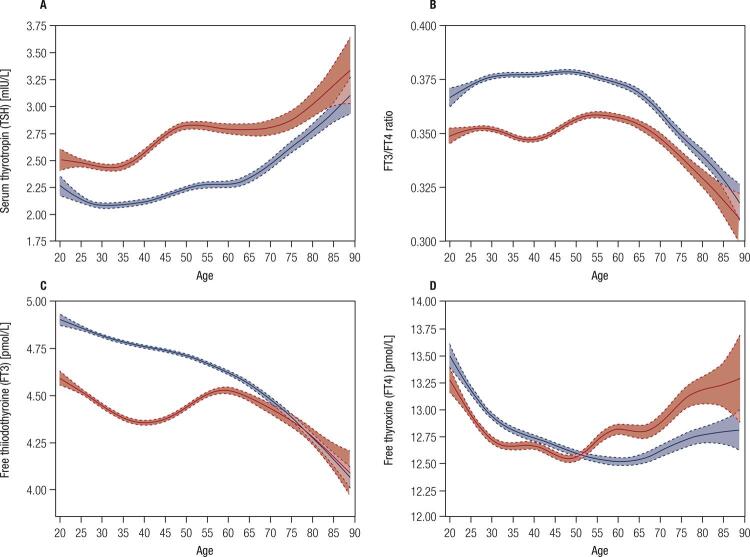
The curve for median TSH, FT3, FT4, and FT3/FT4 ratio vs. age by gender. (A) Shows TSH-age points and smoothing curves for females (red) and males (blue). (B) FT3/FT4 ratios of females (red) and males (blue). (C) FT3 levels of females (red) and males (blue). (D) FT4 levels of females (red) and males (blue).

The intra-assay coefficients of variation (CV) of serum TSH, FT4, FT3, TPOAb, and TgAb were 1.3%–6.3% and the inter-assay CV values were 2.0%-6.6%. The median, 2.5^th^ and 97.5^th^ percentiles of TSH, FT3, FT4 and FT3/FT4 ratio were compared between males and females and the age groups for all participants with negative thyroid antibody (Table 1). The median TSH value increased with age and the median TSH values were the lowest in the 20s and 30s age groups and the highest in the 80s age group for all participants (P < 0.001). Median TSH ranged from 1.98 mIU/L-2.57 mIU/L. Females had a higher TSH value than males (P < 0.001). Accordingly, females had a lower FT3, FT4 and FT3/FT4 ratio in comparison with males (*P* < 0.001). Overall, TSH levels increased with increasing age (Figure 1A). The median TSH increased significantly in females at the age of 30 years. Similar to that observed for the TSH levels, FT3 concentration was higher in males than in females, but a stepwise decrease was noted in FT3 with increasing age (Figure 1C). A similar trend in gender was noted for FT4 levels (Figure 1D). The FT3/FT4 ratio remained stable until age 60 years, followed by a rapid decrease (Figure 1B).

**Figure 2:**
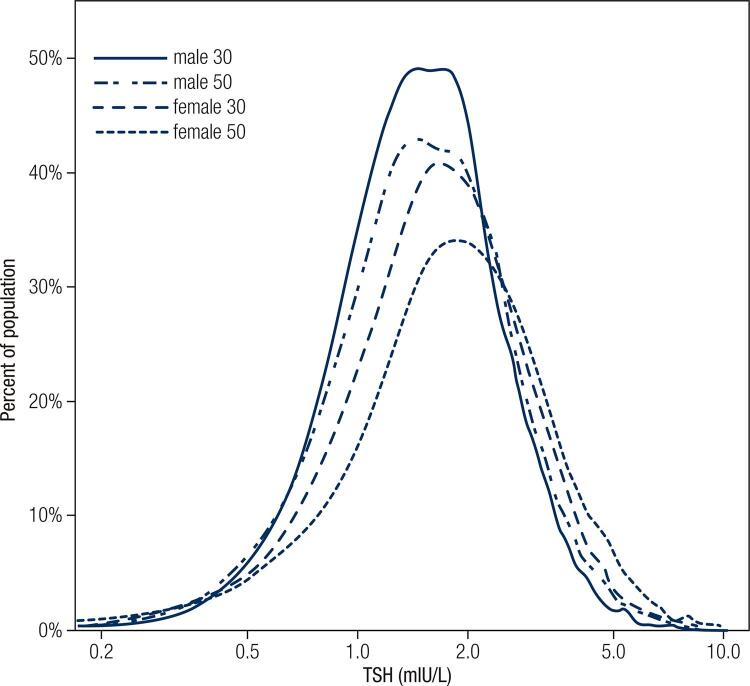
TSH distribution curve for the 30s and 50s age group devided by gender.

The 30s and 50s age groups were selected for comparing the TSH concentration between males and females. As shown in Figure 2, the frequency distribution curves were non-overlapping; the distribution of the 50s age group shifted to higher TSH concentrations and a flat curve was observed in comparison to that in the 30s age group. Differences in the 50s age group were significantly higher than those in the 30s age group (median 2.48 mIU/L for females and 2.00 mIU/L for males in the 50s age group, median 2.18 mIU/L for females and 1.85 mIU/L for males in the 30s age group (*P* < 0.001).

## DISCUSSION

In the present study, the data of 83643 participants without thyroid disease were analyzed and the distribution of median and 2.5th and 97.5th percentiles for TSH, FT3, FT4 and FT3/FT4 ratio were compared by gender and age. We found that TSH values increased with age and that females and older adults (70s age group and over 80s group) had lower FT3 and lower FT3/FT4 ratios, while FT4 level remained stable. Serum thyrotropin increased progressively with age, which agrees with the results of previous studies (3,5,6). Both the females and males showed progressive changes in TSH levels with age. Females had higher TSH levels than males, which was also observed in previous studies (7,15,16). In a retrospective analysis of 465593 TSH measurements from non-thyroid disease subjects, females had a significantly higher TSH than males and the reference intervals for TSH varied significantly by age, gender, time and ethnicity (17). Previous experiments have also suggested that decreases in estrogen may coincide with thyroid hormone activity (18). The Fisher Rat Thyroid Cell Line cells (FRTL-5) contain functional estrogen-responsive reporters (ERs), and estradiol promoted FRTL-5 thyroid cells growth in a time- and concentration-dependent manner, a process occurs in either the absence or presence of TSH (19). Thus, that mechanism may help to explain the gender-specific increase in TSH in premenopausal women. However, the mechanism of increased TSH associated with age after menopause is still not clear, even though the results of the above studies suggest that TSH levels may be associated with gender differences. In the present study, females were shown to have a higher TSH level and a different pattern of TSH increase compared to the males. However, the increase in TSH level associated with age occurred earlier, starting at about age 30 years in females compared with that in males. In a Western Australian study with 148938 individuals, a similar phenomenon was observed but it started at 35-40 years and the difference in TSH levels between females and males was insignificant (20). Suzuki and cols. (21) established a thyroid hormone resistance index based on the log-linear relationship between TSH and free thyroid hormone and proposed a gender-specific response to the relationship of thyroid hormone with age. Their results showed that the resistance indices were not altered in males aged between 25-34 years and 55-64 years, but a tendency for resistance indices to increase was found in the same 25-64 years age groups (21). In 2011, Li and cols. (22) reported that the TSH levels of teens aged 12-19 years were significantly higher than in the other age groups, with no significant differences in the TSH values of other age groups. Although adolescents were not recruited in the present study, a tendency toward increased TSH levels in adults was observed and TSH levels in the 80 years and over age group were significantly higher than those in other age groups.

It appears that the increase in TSH with age may not be secondary to thyroid antibody or iodine alterations. In a longitudinal community-based study conducted in Australia, the age-related TSH increase was thought to stem from age-related alterations in TSH set points or due to reduced bioactivity rather than thyroid disease (23). Genetic factors may also influence these changes, as previously reported (24-26). According to Sun and cols. (27), the upper limit of the reference interval for TSH was increased to 2.38 mIU/L (0.71mIU/L-6.25mIU/L) in the Chinese population. In addition, similar to our results, Yan and cols. (28) reported that the median serum TSH value increased to 2.64 mIU/L in 1334 thyroid disease-free subjects in Chengdu. At the same time, the prevalence of subclinical hypothyroidism in 2012 was reported to be significantly higher compared to that in 1999 (16.7% *vs*. 3.22%) (11). Due to age-related TSH levels, the prevalence of subclinical hypothyroidism would be significantly overestimated unless an age-specific range for TSH was used.

TSH modulates the production of thyroid hormone and is affected by the negative feedback control of thyroid hormone, which is primarily influenced by circulating T3 levels. In the present study, FT3 levels in males showed a stepwise decrease as TSH increased with age. However, FT3 levels in females showed fluctuations in relation to the level of TSH. Interestingly, in FT4 level in both females and males was basically the same. FT3/FT4 ratios remained stable until the age of 60 years followed by a rapid decline. This phenomenon was also reported by Strichet and cols. (29), that is, as TSH increased, the FT3/FT4 ratio increased until the age of 40 years followed by a significant decrease in the 60s age group. In the present study, construction of the age-related curve showed a decrease in FT3 and an increase in TSH – changes seemlingly interdependent of each other. The ratio of serum FT3 concentration to FT4 concentration is a useful indicator for assessing the peripheral metabolism of thyroid hormone, which is affected by the conversion of FT4 to FT3 or due to the transport of T4 into T3-producing tissues (30). The majority of T3 comes from the conversion of T4 in the peripheral circulation, which is regulated by the iodothyronine deiodinases D1, D2 and D3, and iodothyronine deiodinases D2 is the main source of T3 in plasma in euthyroid people (31). Animal studies suggest that serum FT3 and FT4 decrease gradually with age and a significant decrease was shown in the senescent Wistar rats (24 months old), and reduced deiodinase activity may be responsible for this phenomenon (32). Currently, FT3 levels are not recommended by any of the guidelines as criteria for the diagnosis of hypothyroidism. However, the decrease in FT3 seems to be responsible for the increase in TSH in older adults. In this instance, the current accepted normal TSH reference range might not be suitable for the diagnosis of subclinical hypothyroidism in older adults. Biondi and cols. (33) and Atzmon and cols. (34) recommended that individuals with TSH levels above the upper limit of normal should be reassessed every 6-12 months to exclude transient TSH level increase. Individuals with heritable longevity and exceptional longevity have been shown to have higher serum TSH (34-36). Thus, we may speculate that higher serum TSH is beneficial for older adults.

In the present study, the positive definition of thyroid antibodies based on clinical laboratory standards. We excluded antibody positive samples using two anti-thyroid antibodies, which made our analytic sample more rigorous and unified. Nevertheless, this study has some limitations. These include, but are not limited to, medications, dietary habits, pregnancy and menopause.

In conclusion, serum TSH increases with age in the general Chinese population without thyroid disease. In accord with the increased TSH values during aging, females and older adults have lower FT3 values and lower FT3/FT4 ratios, while FT4 values remain stable.
